# Supplementary material for: Sampling locality is more detectable than taxonomy or ecology in the gut microbiota of the brood-parasitic Brown-headed Cowbird (Molothrus ater)
Source: PeerJ. 2014 Mar 20;2:e321. doi: 10.7717/peerj.321 (PMC3970801; doi:10.7717/peerj.321)
Supplement: Supplemental Information 2 — •Supplementary Figure S1. Dendrogram and NMDS for All Louisiana Birds, Cowbirds Only and Hosts Only datasets, with bootstrap scores >0.7 shown.•Supplementary Figure S2. Box plot of UniFrac distances within and between hosts, adult cowbirds and juvenile cowbirds.•Supplementary Figure S3. Relationship between mammal, insect and bird microbiota shown with principal coordinates analysis of unweighted UniFrac distances (left column) and nonmetric multidimensional scaling, using relative abundance of bacterial phyla as input (right column, see Methods). Results are the same as Fig. 5 from the original manuscript, just colored by different attributes. Birds are denoted by circles (cowbirds are black, brood hosts are gray), mammals are squares and insects are triangles. Dashed lines encapsulate all birds, all insects and all mammals. Top row: colors denote taxonomic order of the vertebrate. Bottom row: colors denote dietary specialization: red is carnivorous/mostly animal material. Green is herbivorous/mostly plant material. Blue is omnivorous/mix of plant and animal material. [file peerj-02-321-s002.pdf]

Supplemental Figures – Hird et al.

Sampling locality is more detectable than taxonomy or ecology in the gut microbiota of the brood-parasitic Brown-Headed Cowbird (*Molothrus ater*)

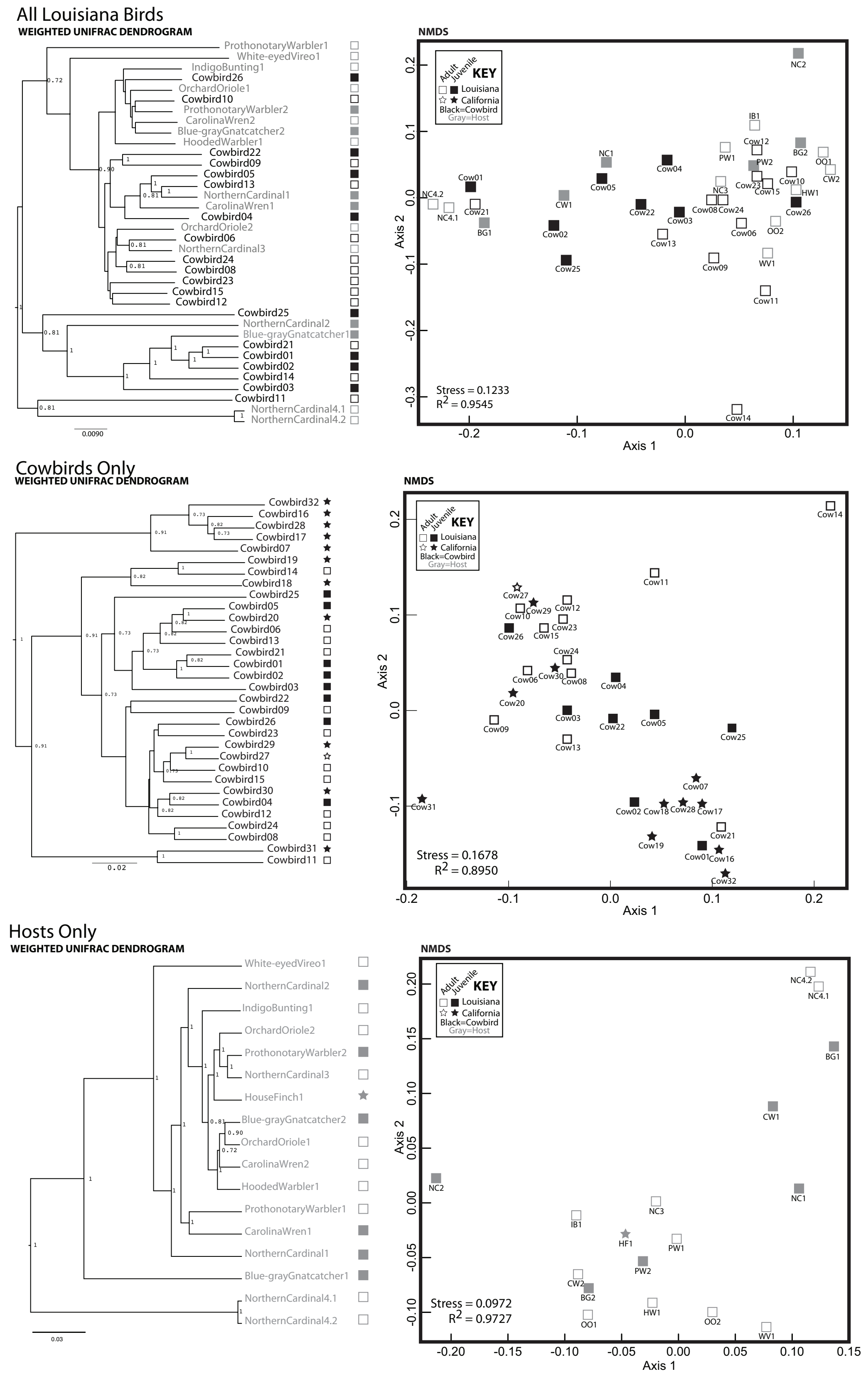

Figure S1: Dendrogram and NMDS for All Louisiana Birds, Cowbirds Only and Hosts Only datasets, with bootstrap scores >0.7 shown.

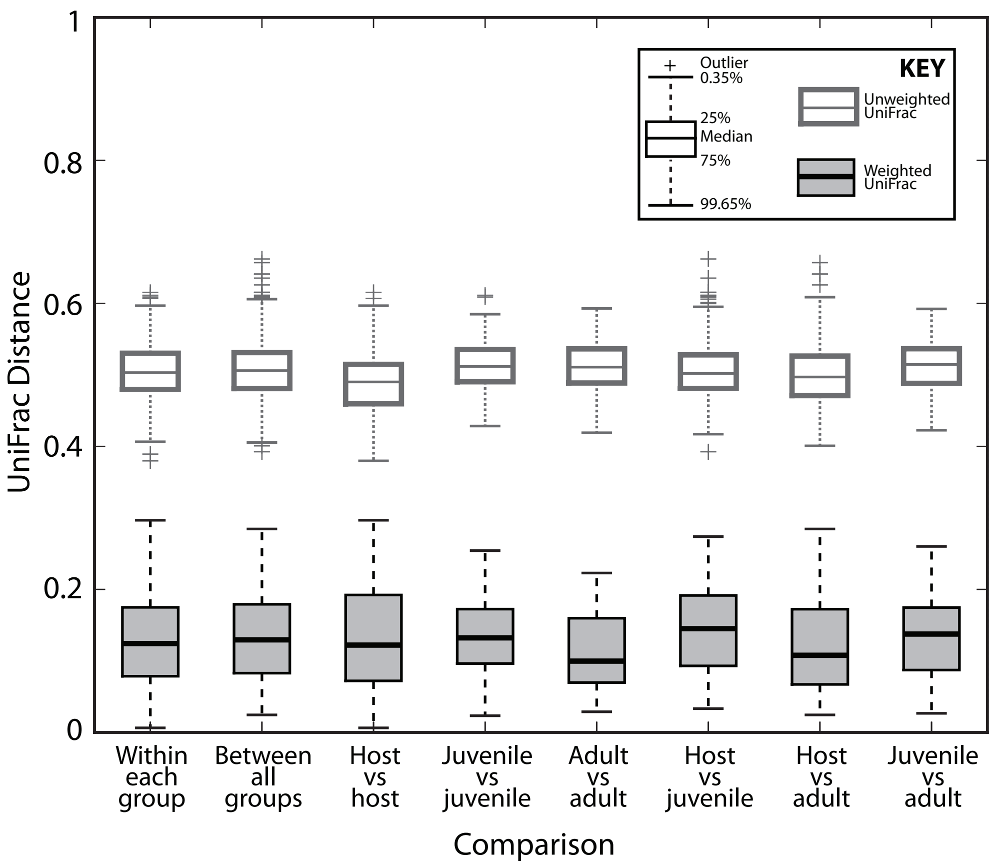

Figure S2: Box plot of UniFrac distances within and between hosts, adult cowbirds and juvenile cowbirds.

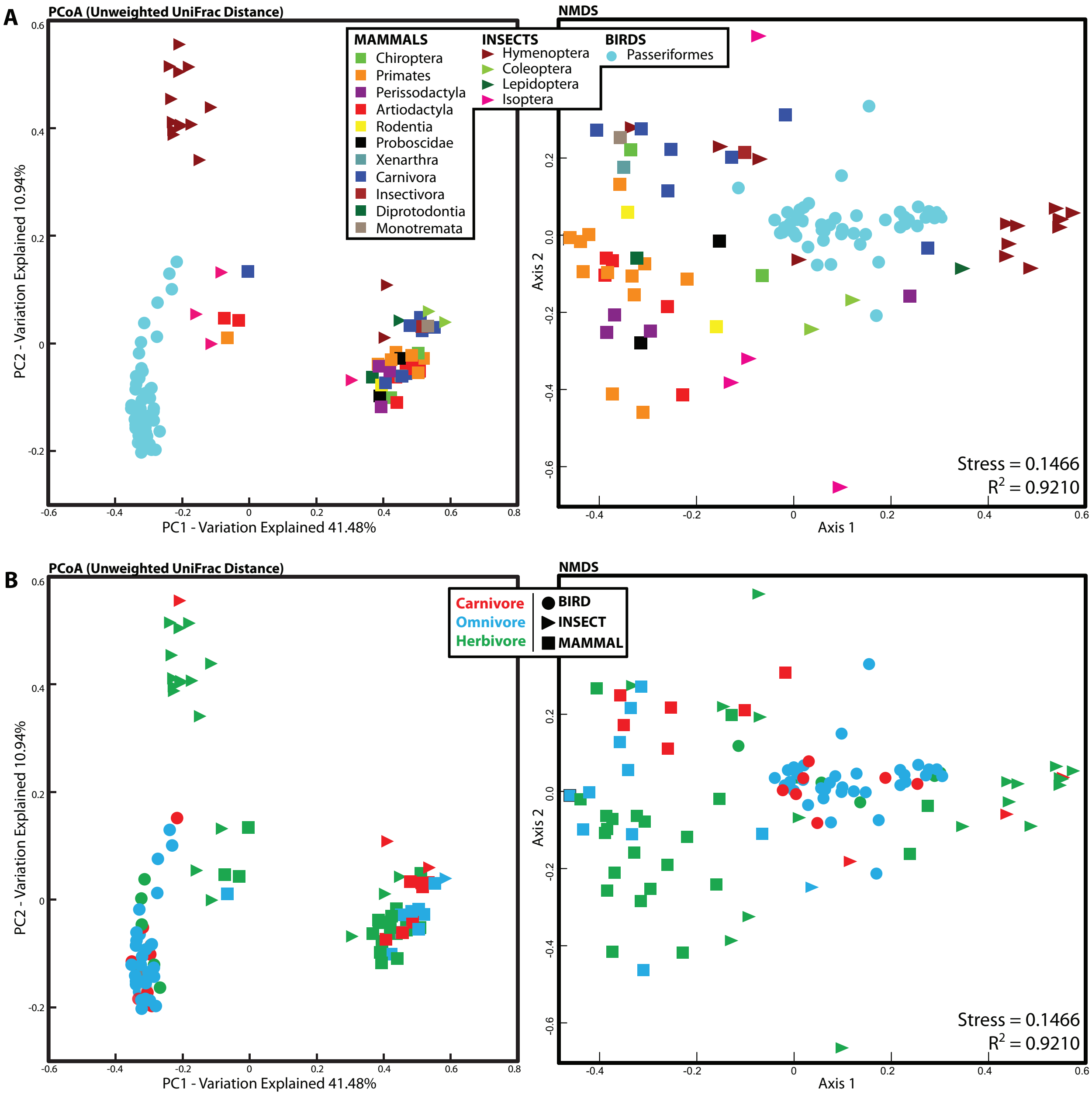

Figure S3: Relationship between mammal, insect and bird microbiota shown with principal coordinates analysis of unweighted UniFrac distances (left column) and nonmetric multidimensional scaling, using relative abundance of bacterial phyla as input (right column, see Methods). Results are the same as Figure 5 from the original manuscript, just colored by different attributes. Birds are denoted by circles (cowbirds are black, brood hosts are gray), mammals are squares and insects are triangles. Dashed lines encapsulate all birds, all insects and all mammals. Top Row: Colors denote taxonomic order of the vertebrate. Bottom Row: Colors denote dietary specialization: Red is carnivorous/mostly animal material. Green is herbivorous/mostly plant material. Blue is omnivorous/mix of plant and animal material.
